# A cognitive model of moral and nonmoral self-criticism: the role of judgment criteria

**DOI:** 10.3389/fpsyg.2026.1824090

**Published:** 2026-09-04

**Authors:** Vittoria Zaccari, Francesco Mancini

**Affiliations:** 1 School of Cognitive Psychotherapy, Rome, Italy; 2 Department of Human Sciences, Guglielmo Marconi University, Rome, Italy

**Keywords:** self-criticism, negative self-evaluation, cognitive model, judgment criteria, moral self-criticism, nonmoral self-criticism, moral ideal self, nonmoral ideal self

## Abstract

Several authors have developed authoritative theoretical models of self-criticism (SC), each offering unique perspectives that emphasize different aspects of its phenomenology. Through a critical examination of these models, this article proposes a new cognitive framework: the *Cognitive model of self-criticism*. The primary aim is to conceptualize the judgment criteria through which individuals evaluate themselves as a central cognitive component of the self-critical process and to provide a theoretical framework for explaining the heterogeneity of SC. The model emphasizes the judgment criteria underlying self-critical evaluations, distinguishing between two types of negative evaluation: moral and nonmoral criteria. This distinction gives rise to two corresponding forms of SC: moral and nonmoral SC. Both forms are characterized by distinct judgment criteria associated with different components, including the emotional tone and attitude of the critic, emotional reactions, expectations, and the behavioral dispositions of the criticized. The model was developed through the integration and extension of previous theoretical conceptualizations, the broader psychological literature, and the cognitive tradition, with particular attention to judgment criteria and the components characterizing the two forms of SC. In addition, the model identifies three further parameters that may help distinguish adaptive from maladaptive expressions of SC: the frequency, intensity, and persistence of self-critical processes; the degree of critical distancing; and the stability vs. contingency of self-critical evaluations. This work introduces a theoretical framework designed to generate empirically testable hypotheses and guide future research on SC. Future empirical studies involving both community and clinical samples will be necessary to examine whether the proposed components reliably differentiate moral and nonmoral forms of SC and to develop a validated assessment instrument. These research directions are intended to support the empirical evaluation and progressive refinement of the model while promoting greater integration between evaluative processes and psychopathological mechanisms.

## 1 Introduction

### 1.1 The role of self-criticism in psychological and psychopathological functioning

Self-criticism (SC) plays a fundamental role in individuals' psychological functioning. It serves an adaptive function in self-monitoring and self-regulation and constitutes a constructive process of considerable importance for personal development. In its adaptive form, SC operates as a self-corrective mechanism, enabling individuals to evaluate their behavior against personal or social standards, recognize mistakes, and implement appropriate changes. It promotes reflexivity and self-awareness by monitoring thoughts, emotions, and actions in the pursuit of valued goals (Gilbert, 2022; Gilbert et al., 2004).

However, in the field of clinical psychology, SC is commonly understood as a process of self-evaluation that is critical, negative, or hostile in nature (Blatt et al., 1976; Blatt, 2008; Gilbert et al., 2004; Shahar, 2015). Indeed, when excessive or pervasive, SC can take on maladaptive forms, contributing to the onset and maintenance of various psychopathological conditions (Barcaccia et al., 2019; Couyoumdjian et al., 2016; Gilbert, 2022; Gilbert and Irons, 2005; Gilbert et al., 2004; Löw et al., 2020; Rainone and Mancini, 2018; Wakelin et al., 2022; Werner et al., 2019). Negative self-evaluation and self-judgment—extending to self-condemnation and self-deprecation—can significantly impair mental health, coping abilities, resilience, and recovery processes (Ehret et al., 2015; Gilbert and Irons, 2005; Mandel et al., 2015; McIntyre et al., 2018; Rogier et al., 2023; Shahar, 2015). The scientific literature highlights the involvement of SC across a wide range of psychopathological disorders, identifying it as a transdiagnostic factor associated with poorer psychotherapy outcomes and increased resistance to treatment (Gilbert and Irons, 2005; Kannan and Levitt, 2013; Löw et al., 2020; McIntyre et al., 2018; Wakelin et al., 2022; Warren et al., 2016; Werner et al., 2019). Maladaptive SC results in emotions such as shame, hatred, disgust, guilt, and anger, as well as adverse emotional states like feelings of inadequacy, failure, and unworthiness (Blatt and Luyten, 2009; Gilbert et al., 2004; Gilbert and Simos, 2022; Shahar, 2015; Whelton and Greenberg, 2005).

Given its clinical relevance, numerous researchers and clinicians have investigated SC and its phenomenology proposing several theoretical frameworks (Blatt et al., 1976; Blatt and Luyten, 2009; Gilbert et al., 2004; Shahar, 2015).

In a recent meta-review, Zaccari et al. (2024a) systematically synthesized the key components through which existing theoretical models conceptualize SC, including the emotional tone of the critic, emotional reactions, behavioral tendencies, and the different forms and functions of SC. The review further highlighted an important theoretical gap, namely the limited attention devoted to the judgment criteria underlying self-critical evaluations.

Building upon this observation, this article conceptualizes judgment criteria as the evaluative standards through which individuals judge themselves during self-critical processes.

Specifically, the present work addresses a question that existing models appear to have left largely unexplored: according to which judgment criterion is the self-evaluated?

### 1.2 A brief overview of theoretical perspectives on self-criticism

The first conceptualization of SC, known as *The Two Polarity Model of Personality Development and Psychopathology*, was developed by Blatt (1974) and by Blatt et al. (1976) within the framework of depression and personality development.

According to this perspective, SC is characterized by inadequacy, guilt, perfectionism, and self-punitive attitudes toward oneself when standards are not perceived to be met (Blatt, 2004; Blatt and Luyten, 2009; Blatt and Zuroff, 1992). Blatt et al. distinguished between two forms of SC: *comparative SC*, which involves comparing our traits or characteristics to those of others, and *internalized SC* (Thompson and Zuroff, 2004), which happens when comparing oneself to what one should ideally be, leading to feelings of not measuring up. Blatt and Zuroff (1992) correlated SC with motives for success and the avoidance of failure through perfectionism.

Shahar (2015) developed a different model called *The Axis of Criticism Model* (ACRIM). This author defined SC as a profound and enduring relationship with oneself, marked by an unwavering expectation of elevated performance standards and a manifestation of hostility and self-deprecation when these elevated standards are inevitably not achieved (Shahar, 2015). Specifically, the model proposes that SC arises from a critical dialogue between two parts of the self, in which one aspect communicates messages of hatred, malice, inadequacy, and deficiency to the other. These messages would have a critical, harsh, and derogatory tone, leading to the development of negative, global, and definitive beliefs or judgments about oneself or one's nature. This would result in negative feelings such as shame, sadness, and anger, reinforcing a distorted self-perception and preventing the activation of positive emotions, such as pride and curiosity, which could instead foster a process of positive self-evaluation. According to Shahar's (2015) theory, one of the fundamental characteristics of SC is its active nature. Self-critical individuals actively create interpersonal conditions—negative events, a lack of positive events, and the absence of social support—that fuel emotional distress, which in turn culminates in an increase in SC. The process would be set up as a vicious cycle known as the “*self-critical cascade*.” The generation of risk factors (negative events) by self-critical individuals is explained through their use of avoidant or maladaptive coping styles.

Gilbert (see Gilbert et al., 2004, 2014) proposed an interesting theoretical conceptualization of SC and distinguished two maladaptive forms of SC: the *Inadequate Self* and the *Hated Self* . The *Inadequate Self* reflects feelings of inferiority, disappointment with oneself, and concerns about personal inadequacy, often accompanied by a constant desire to improve and do better, frequently driven by competitive concerns. Individuals with a prominent *Inadequate Self* tend to focus on perceived weaknesses and shortcomings and experience themselves as unable to meet personal standards or expectations. The *Hated Self* represents a more hostile and punitive form of self-relating characterized by self-disgust, contempt, and self-hatred, rooted in self-loathing where the individual does not seek to improve aspects of themselves but rather wishes to rid themselves of those aspects entirely. Unlike the *Inadequate Self*, which primarily involves feelings of deficiency, the *Hated Self* reflects active self-attack aimed at damaging oneself, marked by disgust, rejection, and a desire to erase a part of oneself—the desire to reject, punish, or eliminate aspects of the self-perceived as unacceptable. Gilbert (2022) described this form of SC as a particularly severe manifestation of internal hostility directed toward the self. In cases of failure or setbacks, the individual may experience frustration, triggering a nonhostile SC regarding their performance, leading to feelings of inadequacy. In contrast, hostile SC, which targets the self rather than performance, according to Gilbert, does not provide corrective solutions for addressing failures. In this case, the inner dialogue of one part of the self expresses hatred, disgust, and contempt toward another part, which responds with intense feelings of shame, a key emotion in Gilbert's theory for understanding SC. Self-hating involves a deep sense of disliking and even hating oneself, leading to a desire to hurt or persecute oneself (Gilbert, 2022; Gilbert et al., 2004).

Alongside these two forms of SC, Gilbert's model also includes the *Reassured Self*, which refers to the capacity to respond to personal failures and difficulties with understanding, support, and self-compassion. *Reassured Self* represents a protective self-regulatory process that may buffer the negative effects of SC and promote psychological resilience (Gilbert, 2014).

Gilbert et al. (2004) highlight the role of a hostile critical tone capable of overwhelming a more compassionate and reassuring aspect of the self. In line with Gilbert's model, Whelton and Greenberg (2005) emphasize that, beyond the content of SC, the tone of the critical voice and the way in which the experiencing self responds—either with resilience or collapse—are particularly relevant.

Additionally, Gilbert maintains that SC is not a single process but involves different forms, functions, and underlying emotions. It can be helpful as a self-correcting and self-monitoring mechanism, providing adaptive feedback by promoting reflection and monitoring of thoughts, emotions, behaviors, or goals. The difference between adaptive and maladaptive SC is linked to the motivation behind, and the emotional negative tone of, the criticism (Gilbert, 2022; Gilbert et al., 2004). It is possible to criticize oneself for things that make one feel inadequate or disappointed, but if these things are not perceived as resulting in negative social consequences, SC does not take on harmful connotations. Therefore, Gilbert emphasizes an important distinction between the helpful and harmful aspects of SC: Forms of criticism based on shame that focus on the sense of an inadequate self (competitive social mentality) are different from criticism related to a caring or cooperative social mentality (Gilbert, 2016), which is based on guilt and focuses on behaviors that have been harmful to others (Gilbert, 2022). In this regard, Gilbert highlights how self-blaming and SC are part of (potentially helpful) self-monitoring and correcting systems.

The way in which people express emotions related to SC, according to Gilbert, can vary. On the one hand, there may be a low tolerance of frustration, where people become angry due to mistakes and feel vengeful anger toward themselves for not meeting their own expectations. Others experience anxiety or anger because a failure or setback triggers a deeper sense that they could (and should) be better if only they could do things in the right way. In these cases, they feel they have disappointed themselves due to a poor performance. In other individuals, the emotion experienced is self-disgust, where they perceive certain aspects of themselves as alien and wish to eliminate them.

With regard to the implications of SC or the coping strategies employed, Gilbert notes that people who hate themselves strike out and harm themselves in many ways (Gilbert et al., 2010), predominantly using self-destructive and self-punishing strategies. SC linked to self-hatred and the desire to hurt and persecute oneself leads to feelings of being undesirable and can push individuals into a state of disconnection, but not necessarily into striving for competitiveness or defending themselves through avoidant and/or submissive strategies.

### 1.3 Theoretical expansion of self-criticism: introducing judgment criteria as an additional cognitive component

The distinctions among the different forms of SC proposed across various conceptualizations represent a crucial contribution to understanding the phenomenon and its role in psychopathology. All theoretical frameworks highlight the importance of considering certain fundamental aspects in order to comprehend the nature and functions of SC—namely, the emotional tone, the emotional reaction to criticism, and the implications for action and behavioral tendencies. Together, these components provide important information about the underlying evaluative nature of self-critical judgments.

Throughout the present paper, we use the term judgment criteria to refer to the evaluative standards through which individuals assess their own actions, thoughts, emotions, and personal characteristics.

Miceli and Castelfranchi (2000) distinguish three ways in which an evaluation can be produced: by performance, when the evaluating agent directly tests whether something favors a goal; by classification, when an evaluation is inherited from the class an entity belongs to; and by standard, when the agent explicitly represents the set of qualities that make a class of entities good for a given goal, and assesses whether a specific instance possesses those qualities. The judgment criteria we refer to throughout this paper correspond to this latter notion of evaluative standard: the set of qualities a person implicitly attends to when judging themselves. Just as one might judge a pair of scissors as good by checking whether it has the right qualities for cutting, a person judging themselves checks whether they possess the qualities required by a given standard: nonmoral standards (competence, ability, desirability) or moral standards (responsibility, harmlessness).

This perspective is also consistent with appraisal theories of emotion (Arnold, 1960; Ellsworth, 2013; Frijda, 1986; Frijda et al., 1989; Lazarus, 1991; Ortony et al., 1988; Roseman and Smith, 2001; Scherer, 2001, 2009), according to which emotions are not determined directly by events themselves but by the appraisal processes through which individuals evaluate those events. Since SC is itself a form of self-evaluation, it necessarily involves appraisal processes through which individuals assess themselves against evaluative standards. From this perspective, judgment criteria can be understood as organizing the evaluative appraisal through which individuals judge themselves. Consequently, different judgment criteria may organize qualitatively different forms of self-appraisal, with corresponding emotional and behavioral implications.

Taken together, these theoretical perspectives converge in suggesting that self-critical judgments necessarily involve evaluative standards.

However, existing theoretical models appear to refer to such standards only implicitly and do not explicitly conceptualize them as an autonomous cognitive component of self-critical evaluation. As we will show in the following sections, it is possible to distinguish, within existing models, between an implicit presence of such criteria—traceable, through some interpretive effort, in some of them—and their explicit treatment as an autonomous organizing principle. It is the latter, rather than the former, that is absent across available models.

Further support for this conceptualization comes from the cognitive–behavioral perspective, particularly from the notion of mental state (Dimaggio and Semerari, 2003) and the ABC model (Beck, 1976; Beck and Haigh, 2014; Ellis, 1962, 1996, 2001), which emphasize how evaluative beliefs and standards influence emotional and behavioral responses (Beck, 2011; Clark and Beck, 2010). Within cognitive therapy, therapeutic interventions—particularly through cognitive restructuring—primarily focus on modifying the evaluative beliefs and standards through which individuals interpret and judge themselves, rather than directly targeting emotional reactions such as shame, guilt, avoidance, or reparative behaviors (Beck, 1976; Beck and Haigh, 2014; Ellis, 1962, 1996, 2001). From this perspective, changes in these evaluative standards are assumed to contribute to corresponding changes in emotional experience and behavioral responses.

Every act of SC necessarily presupposes an evaluative standard against which the self is judged. Without such an evaluative standard, no self-critical judgment can logically exist, since every judgment necessarily presupposes both an object of evaluation and a criterion against which that object is assessed. Emotional reactions and action tendencies may emerge as downstream consequences of the evaluative standard that is activated.

These considerations suggest that judgment criteria constitute an additional cognitive component of the self-critical process that functions as an autonomous organizing principle of self-critical evaluation. As such, they provide the cognitive foundation through which self-evaluative judgments are organized and help explain why SC manifests in qualitatively different ways and gives rise to distinct emotional and behavioral patterns. This perspective motivates a closer examination of how existing theoretical models have conceptualized judgment criteria, whether explicitly or implicitly, and the extent to which they have recognized their organizing role within the self-critical process.

### 1.4 Judgment criteria across existing models of self-criticism

The following overview considers the principal theoretical models of SC from this perspective, asking whether the evaluative standards underlying self-critical judgments are explicitly conceptualized or can only be inferred from their theoretical formulations.

In Blatt's model, the two forms of SC conceptualized—one characterized by the tendency to compare one's own characteristics with those of others and the other focused on comparing oneself to an idea of what one thinks they should be—do not clarify whether the comparison with others always involves using the same criteria that are applied to self-judgment. The two forms of SC may rely on the same judgment criterion, although this possibility is not made explicit in Blatt's original formulation.

In the first case, self-evaluation is relative to the judgment made about others (*am I better or worse?*), while in the second case, self-evaluation may occur according to the same criteria, but without direct reference to others. Notably, what may change across these two forms is therefore not necessarily the nature of the criterion itself, but rather the standpoint from which it is applied: in one case, the comparison is external, relative to the discrepancy with others; in the other, it is internal, defined with reference to one's own ideal. However, both interpersonal and self-ideal-based SC may rely on the same judgment criterion: for example, I judge myself a failure for getting a bad grade in an exam, and according to my internal standards, the grade is too low, or I judge myself a failure because I got a lower grade than a colleague. In both cases, the judgment criterion is the same: the school grade, while only the standpoint of comparison (internal vs. external) differs. This interpretation suggests that, although not explicitly theorized, judgment criteria may represent an implicit and unifying component underlying Blatt's distinction between comparative and internalized forms of SC.

With respect to the ACRIM, Shahar emphasizes that in SC, one part of the self tells another not only that it is lacking or inadequate but also that it is bad: “*You are generally bad*,” “*generally deficient*” (Shahar, 2015, p. 78). We argue that Shahar's conceptualization can be interpreted as implicitly pointing to two distinct judgment criteria—deficiency and badness—each representing a different evaluative standard. Specifically, deficiency would correspond to a competence-based criterion, whereas badness would reflect a moral or ethical one. This interpretation suggests a qualitative difference between these forms of SC, attributable to the type of evaluative standard applied in the self-critical process. From this perspective, Shahar's model appears to distinguish different judgment criteria implicitly, although it does not conceptualize them as an autonomous component of self-critical evaluation.

Gilbert's model highlights how SC can occur both on issues of inadequacy and by attacking the “global self” through a hostile tone, which would exist in a severe form of SC. This model emphasizes that criticism can be expressed in two different ways: directed at performance or at the global self. However, it does not explicitly address the judgment criteria underlying these forms of criticism, namely the evaluative standards used to assess the self. Gilbert's distinction can be interpreted as being compatible with different judgment criteria rather than as specifying the evaluative standards themselves. Indeed, the same target of criticism—whether performance or the global self—may be evaluated according to different judgment criteria. For instance, a hostile attack on the global self may stem either from perceived incompetence or deficiency relative to standards of competence and achievement, or from perceived harmfulness, moral badness, or the transgression of a moral rule. Gilbert's distinction between forms and functions of SC therefore operates at a level of analysis that is complementary to, rather than overlapping with, the judgment criteria proposed in the present paper.

These models appear to contain implicit references to evaluative standards; however, none explicitly conceptualizes judgment criteria as an autonomous organizing principle of self-critical evaluation.

### 1.5 Moral and nonmoral nature of judgment criteria

These theoretical reflections reveal that a crucial element is still lacking for a clear differentiation among the various forms of self-critical internal dialogue—namely, the judgment criterion through which individuals evaluate themselves. Whereas the theoretical models reviewed above primarily describe SC in terms of its emotional, interpersonal, functional, or behavioral characteristics, the present proposal focuses on the evaluative standard through which the self is judged.

Importantly, the present proposal should not be interpreted as replacing or reducing the relevance of emotional tone, emotional reactions, or interpersonal dynamics emphasized by previous models. Rather, judgment criteria are proposed as a complementary cognitive dimension that operates at a different level of analysis, specifying the evaluative standard through which self-critical judgments are generated.

More specifically, we propose that judgment criteria can be organized around a fundamental distinction between what we term moral and nonmoral judgment criteria, a distinction we specify in detail below. This distinction, we argue, constitutes the organizing principle through which two qualitatively different forms of SC emerge, depending on whether self-evaluation is guided primarily by moral or nonmoral standards.

Since the 1930s, psychologists have shown considerable interest in the ontogenesis of moral judgment. Influential classical theories have placed at the center the question of what constitutes a good person, a just norm, or a right action (Kohlberg and Kramer, 1969; Margoni, 2018; Margoni and Surian, 2017, 2020; Piaget, 1965), thereby paving the way for recognizing the importance of moral standards in self-evaluation processes (Benson, 2001). The psychological tradition in the study of moral standards has evolved from Freudian psychoanalysis to contemporary frameworks, progressively deepening our understanding of how individuals develop and apply moral standards in evaluative processes, with particular emphasis on the emotion of guilt (Carnì et al., 2013). Contemporary theories of emotion and moral cognition recognize that both moral and nonmoral standards guide self-evaluation.

These standards are related to behaviors, goals, beliefs, or traits for which individuals feel personally responsible and potentially harmful—unlike nonmoral standards, which pertain to the nonmoral ideal self and concern one's adequacy in relation to personal desires or aspirations (Miceli and Castelfranchi, 2018).

In line with the theoretical perspective developed by Miceli and Castelfranchi (2018), individuals may criticize themselves not only for perceived incompetence or inadequacy but also for their perceived immoral nature when evaluated against moral, ethical, or deontological standards, which together define what we term the moral ideal self.

The authors emphasized that SC can take different forms in internal dialogue: Individuals may perceive the self as (a) ugly, stupid, or deficient—lacking in physical attractiveness, intelligence, or other desirable qualities, or as (b) morally flawed, evil, unjust, or sinful —possessing the ability to violate norms and being willing (or inclined) to do so. Accordingly, self-critical processes may be organized around qualitatively different judgment criteria, depending on whether the evaluation concerns perceived inadequacy or perceived moral transgression.

Thus, the self can be evaluated negatively when certain goals are obstructed or compromised, either through negative appraisals related to inadequacy or helplessness, or through evaluations of harmfulness or malice (Castelfranchi, 1988; Castelfranchi and Parisi, 1980; Conte and Castelfranchi, 1995). According to cognitive theory and cognitive science (Miceli and Castelfranchi, 2000), these judgments can be distinguished according to the criterion underlying the evaluation: on the one hand, a negative evaluation may concern a perceived lack of power—that is, the entity's insufficiency or inadequacy in favoring a given goal; on the other hand, it may concern a perceived negative power—that is, the entity's capacity to actively frustrate or harm that goal. These two forms of negative evaluation are qualitatively distinct, as powerlessness differs fundamentally from any form of negative power, and they are typically associated with different attitudes and emotional reactions on the part of the evaluator (Miceli and Castelfranchi, 2000). Within this framework, perceived responsibility and perceived power are conceptually related, as responsibility presupposes the perceived capacity to intentionally influence one's actions and their consequences. Consequently, moral self-evaluations are typically organized around the appraisal of possessing the power to violate moral standards, whereas nonmoral self-evaluations are more often organized around the appraisal of lacking the power to attain desired standards or goals (Miceli and Castelfranchi, 2018).

As a result, self-critical internal dialogue may be directed toward either the nonmoral ideal self or the moral ideal self. Individuals may therefore experience self-hatred (Gilbert, 2022) either because they perceive themselves as inadequate relative to their nonmoral ideal self or because they perceive themselves as morally bad relative to their moral ideal self.

SC may therefore be directed toward two distinct forms of ideal self: the nonmoral ideal self and the moral ideal self, regardless of whether the internal dialogue is shaped by comparisons with others or with one's own ideals.

Nonmoral and moral self-evaluation can both be understood as involving a perceived discrepancy between the actual self and an ideal self: nonmoral self-evaluation concerns a discrepancy with the nonmoral ideal self (grounded in standards of competence, ability, or desirability), whereas moral self-evaluation concerns a discrepancy with the moral ideal self (grounded in moral standards) (Miceli and Castelfranchi, 2018).

It is therefore possible to hold a negative self-evaluation in relation to one's own moral standards—that is, standards tied to behaviors, goals, beliefs, or traits perceived as harmful, for which one feels personally responsible. This perspective involves a self-evaluation centered on harm or irresponsibility, namely a moral transgression.

Accordingly, negative self-evaluation may be organized around two qualitatively different judgment criteria. One concerns perceived inadequacy in meeting the standards of the nonmoral ideal self; the other concerns the perception of oneself as possessing both the power and the willingness to cause harm or violate internalized moral standards—that is, the standards of the moral ideal self (Mancini and Gangemi, 2021; Miceli and Castelfranchi, 2018).

Importantly, this distinction is intended not merely as a classification of self-critical experiences but as an explanatory framework. Specifically, we propose that the judgment criterion organizing self-evaluation helps explain why phenomenologically similar forms of SC may arise from qualitatively different evaluative standards.

Phenomenologically similar self-critical experiences do not necessarily reflect the same judgment criterion. The judgment criterion is not defined by the phenomenological content of self-evaluation itself, but by the evaluative standard through which the self is judged. Thus, the same self-description (e.g., “*I am ugly*”) may be organized around a nonmoral judgment criterion when the internal dialogue is organized around standards of competence, adequacy, or desirability (e.g., “*I lack the qualities I wish I had*”). Conversely, the same self-description may be organized around a moral judgment criterion when the internal dialogue is organized around standards of moral responsibility, harmlessness, or moral norm violation (e.g., “*I have violated an important moral norm*” or “*I have become a bad person because of what I have done or thought*”). Similarly, the experience of feeling “*broken*” may reflect either a perceived discrepancy relative to one's nonmoral ideal self or the appraisal of having become morally corrupted following the violation of an internalized moral norm. By contrast, describing oneself as “*a sinner*” more directly reflects self-evaluation organized around a moral judgment criterion.

The proposed distinction should not be interpreted as implying that moral and nonmoral judgment criteria are invariably mutually exclusive. Different evaluative standards may simultaneously contribute to the organization of self-evaluation. The purpose of the proposed framework is to identify the evaluative standard that primarily organizes self-evaluation within a given self-critical episode.

Having introduced the distinction between moral and nonmoral judgment criteria, the following section examines how these different evaluative standards contribute to qualitatively different self-critical emotional experiences.

### 1.6 Emotional reactions associated with moral and nonmoral judgment criteria

As reflected in the different theoretical models, the various conceptualizations diverge in how they represent the affective experience of the self-critical individual. These experiences range from feelings of inadequacy to the sense of being “bad” (Zaccari et al., 2024a). Blatt's model describes different types of emotional reactions: Individuals may experience a fear of failure and feelings of unworthiness (i.e., perceiving themselves as failures or as inadequate in comparison to others or to their own standards), as well as guilt and shame, and Shahar's model further expands this perspective to include feelings of deficiency, inadequacy, and badness, and emotions such as shame, sadness, and guilt. For Gilbert, alongside feelings of inadequacy, shame plays a central role in the self-critical process, characterizing its more pathogenic forms. Notably, only Shahar identifies the feeling of “wickedness.”

In our view, the distinction between feelings of inadequacy and wickedness deserves particular attention, as it suggests that these emotions may reflect different judgment criteria underlying the self-critical process. A similar consideration applies to guilt and shame, which may emerge as distinct outcomes of SC depending on the evaluative criterion involved.

The present proposal focuses on the distinction between shame and guilt, recognizing both as self-critical emotions (Miceli and Castelfranchi, 2018). These are generally recognized as self-conscious emotions that emerge from critical self-evaluation and are characterized by negative feelings associated with perceived shortcomings or transgressions (Tangney and Dearing, 2002; Tangney and Tracy, 2012; Tangney et al., 2007).

However, despite broad agreement on their central role in self-evaluation, the criteria used to distinguish shame from guilt remain debated.

Among the most influential accounts, Lewis (2000) and Tangney and Dearing (2002; Tangney et al., 2007) distinguish shame and guilt primarily according to the object of self-evaluation, proposing that shame involves a negative evaluation of the global self, whereas guilt concerns specific behaviors.

Indeed, although there are numerous parallels between shame and guilt, several authors have emphasized that they are distinct emotions, highlighting their differences: shame specifically pertains to nonmoral errors, including issues arising from physical imperfections, incompetence, or inadequacy, for which the individual bears no responsibility, whereas guilt is elicited by moral violations and encompasses errors for which one feels accountable (e.g., Sabini and Silver, 1997; Smith et al., 2002).

Within the cognitive appraisal tradition, Lazarus (1991) proposed a partially different perspective, distinguishing shame as the failure to live up to an ego-ideal from guilt as the appraisal of having transgressed a moral imperative. Although Lazarus did not formulate this distinction in terms of judgment criteria, his account already points toward two qualitatively different evaluative standards.

Building upon this appraisal perspective, the present proposal focuses on the judgment criterion through which the self is evaluated as central to the organization of self-critical emotional experiences.

We propose that self-evaluations organized around standards of competence, adequacy, or desirability are more likely to be associated with shame, whereas self-evaluations organized around standards of moral responsibility, harmlessness, or norm violation are more likely to be associated with guilt, particularly deontological guilt. This proposal does not imply a one-to-one correspondence between judgment criteria and emotions; rather, it suggests that different judgment criteria are more likely to organize different predominant emotional configurations.

According to Miceli and Castelfranchi (2018), shame and guilt are both unpleasant emotions that involve a negative self-evaluation in comparison to one's own standards; nevertheless, they differ in terms of certain self-evaluative components and judgmental criteria. Shame has been defined as an unpleasant feeling that involves a self-evaluation of one's insufficiency in comparison to one's nonmoral ideal self. When someone is ashamed, they could think that they are either guilty or not of a wrongdoing. Individuals may experience shame regardless of whether they consider themselves guilty of a wrongdoing.

Shame is often associated with guilt but is elicited on very specific grounds. Indeed, when individuals experience shame, they are primarily preoccupied with the disappointing discrepancy between their ideal (excellent) and actual (not-so-good) selves, rather than with questions of responsibility. This feeling arises from a compromise between the ideal self and the underlying drive to preserve a positive self-image (Miceli and Castelfranchi, 2018; Rozin et al., 1999; Sabini and Silver, 1997).

Shame is defined as a nonmoral emotion that involves the nonmoral or “aesthetic” aspect of self-esteem, concerning the adequacy of the self in relation to one's desires or aspirations (Miceli and Castelfranchi, 2018). Contrarily, guilt is defined as an uncomfortable feeling that involves negative evaluations of oneself in relation to one's moral standards—that is, the standards pertaining to behaviors, actions, goals, beliefs, and characteristics for which one holds oneself responsible. Guilt implies a self-evaluation of harm or irresponsibility—that is, of wrongdoing—and the appraisal is negative since such actions and goals are viewed as harmful. Wrongdoing can be real or potential, arising from personal traits perceived as modifiable through effort, with the person feeling responsible for not attempting to change them. Thus, guilt involves not only behavior but also the self (Miceli and Castelfranchi, 2018). Importantly, contemporary cognitive theories distinguish between altruistic guilt and deontological guilt (Haidt et al., 2000; Sunstein, 2005; Mancini and Gangemi, 2021). Whereas altruistic guilt arises from the appraisal of having caused harm—or of having failed to care adequately—for another person, deontological guilt derives from the perception of having violated an internalized moral norm, even in the absence of a victim or any concrete harm. Thus, moral self-evaluation extends beyond the appraisal of interpersonal harm and encompasses violations of internalized moral standards. This distinction is particularly relevant to the present proposal because it suggests that the moral judgment criterion should not be reduced to the evaluation of harm inflicted on others but also includes the appraisal of having transgressed deontological obligations.

Consistent with this moral/aesthetic distinction, Miceli and Castelfranchi (2018) further indicate that the negative self-evaluations linked to guilt and shame both relate to the self, though within these two separate domains: individuals who feel guilty perceive themselves as morally reprehensible and engage in self-reproach, whereas those who experience shame regard themselves as deficient or lacking in physical, intellectual, or moral aspects, leading to self-dislike.

Consequently, shame suggests the sense of an inability to fulfill the expectations of one's nonmoral ideal self, whereas guilt indicates the perception of possessing the power and willingness either to cause harm or to violate internalized moral standards, thereby transgressing the standards of one's moral ideal self. This interpretation is also consistent with the literature on the Social Cognitive Chain of Being (Kelman, 1976; Opotow, 1990, 2001; Haslam, 2006; Brandt and Reyna, 2011), according to which individuals implicitly represent persons within a hierarchy of moral worth. From this perspective, violating a fundamental moral norm may be experienced as a fall in one's moral rank, leading individuals to infer that they have become morally diminished—that is, morally corrupted, contaminated, or unworthy—and therefore no longer corresponding to their moral ideal self.

Likewise, the *Do Not Play God* principle (Sunstein, 2005) and the conceptualization of deontological guilt proposed by Mancini and Gangemi (2021) suggest that merely violating an internalized moral norm may lead individuals to infer that they have become morally bad or unworthy, even in the absence of a victim or any concrete harm. From this perspective, guilt—and particularly deontological guilt—may also involve a global negative evaluation of the self.

Accordingly, within the theoretical framework adopted in the present work, the distinction between shame and guilt is not primarily grounded in whether self-evaluation is global or behavior-specific, but rather in the judgment criterion organizing that evaluation.

Finally, it is worth acknowledging that part of the apparent overlap between shame and guilt reported in the literature may also reflect linguistic and cultural factors, in addition to theoretical differences. Cross-cultural studies suggest that the English term *shame* encompasses a broader semantic field than its Italian (*vergogna*) and Spanish (*vergüenza*) counterparts (Hurtado de Mendoza et al., 2010). Whereas in English *shame* is frequently used to refer both to experiences involving social exposure and to experiences involving moral transgression, the Italian *vergogna* and the Spanish *vergüenza* are more strongly associated with social exposure, embarrassment, and appearing ridiculous in the eyes of others. This semantic asymmetry may partly contribute to the apparent overlap between shame and guilt reported in the literature. Indeed, recent studies have highlighted that, in the American context, shame and guilt tend to be more intertwined, whereas this overlap is not observed in the Italian population (Giorgetta et al., 2023).

From this perspective, cross-cultural semantics do not replace existing psychological theories but provide an additional rationale supporting the distinction between the judgment criteria underlying self-critical experiences.

Overall, the present proposal suggests that judgment criteria help explain why qualitatively different emotional reactions may emerge from different forms of self-evaluation.

### 1.7 Behavioral reactions associated with moral and nonmoral judgment criteria

Although existing theoretical models have described a wide range of behavioral reactions associated with SC, they have not clearly specified how these behavioral tendencies emerge from different judgment criteria underlying self-critical evaluation. Nonetheless, previous models consistently suggest that different forms of SC are associated with distinct coping strategies and behavioral responses (Zaccari et al., 2024a). Across models, several behavioral tendencies have been identified, including perfectionistic striving, hyper-compensatory behaviors aimed at success or error avoidance, avoidance strategies, submissive behaviors, self-punitive or self-destructive actions, and other maladaptive coping mechanisms (Blatt, 2004; Blatt and Luyten, 2009; Blatt and Zuroff, 1992; Gilbert et al., 2010; Shahar, 2015).

On this basis, different judgment criteria are proposed to organize qualitatively different motivational and behavioral tendencies. More specifically, SC directed toward the immoral or bad self, arising from the appraisal of having violated the standards of the moral ideal self, is more likely to organize guilt-related emotional configurations. These emotional experiences typically motivate reparative actions, expiation, confession, self-punitive behaviors, compulsive or ritualized behaviors, reassurance seeking, and other attempts aimed at restoring one's moral integrity and reducing perceived moral responsibility (Miceli and Castelfranchi, 2018).

Conversely, SC directed toward the deficient or inadequate self, arising from the perceived discrepancy between the actual self and the nonmoral ideal self, is more likely to organize shame-related emotional configurations. These emotional experiences typically motivate withdrawal, avoidance, submissive behaviors, perfectionistic striving, hyper-compensatory efforts directed toward achievement or error prevention, and other strategies aimed at restoring competence, adequacy, or a desired self-image (Miceli and Castelfranchi, 2018).

Overall, the proposed model suggests that self-critical judgment is not limited to evaluating whether individuals are adequate or inadequate, but also concerns whether they evaluate themselves as morally bad or morally responsible. From this perspective, the different behavioral tendencies identified by previous theoretical models can be understood as organized by qualitatively different judgment criteria rather than as independent manifestations of SC.

### 1.8 The emotional tone of the critic and the expectations of the criticized associated with moral and nonmoral judgment criteria

Although previous theoretical models have described different emotional tones characterizing the self-critical voice, they have generally treated these as descriptive characteristics of SC rather than as consequences of different evaluative standards (Zaccari et al., 2024a). Blatt and Shahar primarily describe critics as punitive, harsh, judgmental, and deprecative, whereas Gilbert further emphasizes their hostile, hateful, disgusted, coercive, and attacking nature, proposing that these emotional qualities reflect activation of the threat system rather than adaptive self-correction (Gilbert and Simos, 2022). Similarly, Shahar (2015) characterizes the self-critical voice as an uncompromising prosecutor engaged in a relentless internal trial.

Within the present framework, we propose that different emotional tones may likewise be understood as expressions of the judgment criterion organizing self-evaluation. When SC is organized around nonmoral judgment criteria, the critic is more likely to adopt a disqualifying, ridiculing, or humiliating tone directed toward the deficient or inadequate self, with the aim of highlighting perceived incompetence or inadequacy relative to the nonmoral ideal self. Conversely, when SC is organized around moral judgment criteria, the critic is more likely to adopt a condemning, blaming, accusatory, or contemptuous tone directed toward the immoral or bad self, reflecting the appraisal of moral wrongdoing and the need for moral condemnation or expiation.

These differences may be more readily appreciated through two brief clinical illustrations based on phenomenological reconstructions of the inner critic as reported by patients during psychotherapy:

*Marc*, a 32-year-old patient with social anxiety disorder, is invited during psychotherapy to reconstruct the dialogue of his inner critic immediately before giving a presentation at work. He reports the following internal dialogue: “*Look at yourself. You're completely incompetent. Everyone will realize that you don't know what you're talking about. You'll embarrass yourself, as always. You'll never be as capable as your colleagues. You're simply not good enough. They'll think you're ridiculous and completely inadequate*.” Marc describes this inner critic as humiliating, ridiculing, disqualifying, and relentlessly pressuring, conveying the message that he is fundamentally inadequate and incapable of meeting the standards of his nonmoral ideal self. The predominant emotional reaction is shame, one of the central emotions in social anxiety disorder (Clark and Wells, 1995; Wells, 1997), accompanied by feelings of inadequacy and inferiority. His predominant expectation is that others will ridicule, reject, humiliate, or socially exclude him because of his perceived inadequacy.

By contrast, *Sophia*, a 35-year-old patient with obsessive-compulsive disorder characterized by intrusive unwanted sexual thoughts involving children, is invited during psychotherapy to reconstruct the dialogue of her inner critic immediately after experiencing one of these intrusive thoughts. She reports the following internal dialogue*:* “*How could you even think something like that? How disgusting you are! How could you even think something like this? What kind of person are you, to have thoughts like that! You shouldn't have allowed yourself. You're a perverse and disgusting person. You're immoral. Deep down, this is who you really are. Normal people don't have thoughts like yours. You deserve to be condemned. You're unforgivable*.” *Sophia* describes this inner critic as condemning, blaming, accusatory, contemptuous, and relentlessly prosecuting, conveying the message that she is fundamentally immoral and deserving of blame. The predominant emotional reaction is deontological guilt (Mancini, 2018; Mancini and Gangemi, 2021), one of the core emotional experiences associated with obsessive-compulsive disorder, accompanied by an urgent need to neutralize the perceived moral threat through checking, mental rituals, reassurance seeking, confession, and other compulsive or reparative behaviors (Semeniuc and Soponaru, 2022). Her predominant expectation is that she deserves blame, condemnation, and punishment because she perceives herself as morally bad.

Together, these clinical illustrations show how different judgment criteria organize coherent configurations of the emotional tone of the critic, predominant emotional reactions, expectations, and behavioral tendencies. In Marc's case, the self is evaluated against a nonmoral ideal self, giving rise to a humiliating and disqualifying critic, shame, and expectations of ridicule and social exclusion. In Sophia's case, the self is evaluated against a moral ideal self, giving rise to a condemning and blaming critic, deontological guilt, and expectations of blame, condemnation, and punishment. From this perspective, the emotional tone of the critic represents another manifestation of the judgment criterion underlying self-critical evaluation and contributes, together with emotional reactions, expectations, and behavioral tendencies, to organizing qualitatively different forms of SC.

## 2 Theoretical proposal on self-criticism: cognitive theoretical model

### 2.1 Components of the cognitive model of moral and nonmoral self-criticism

**Figure 1:**
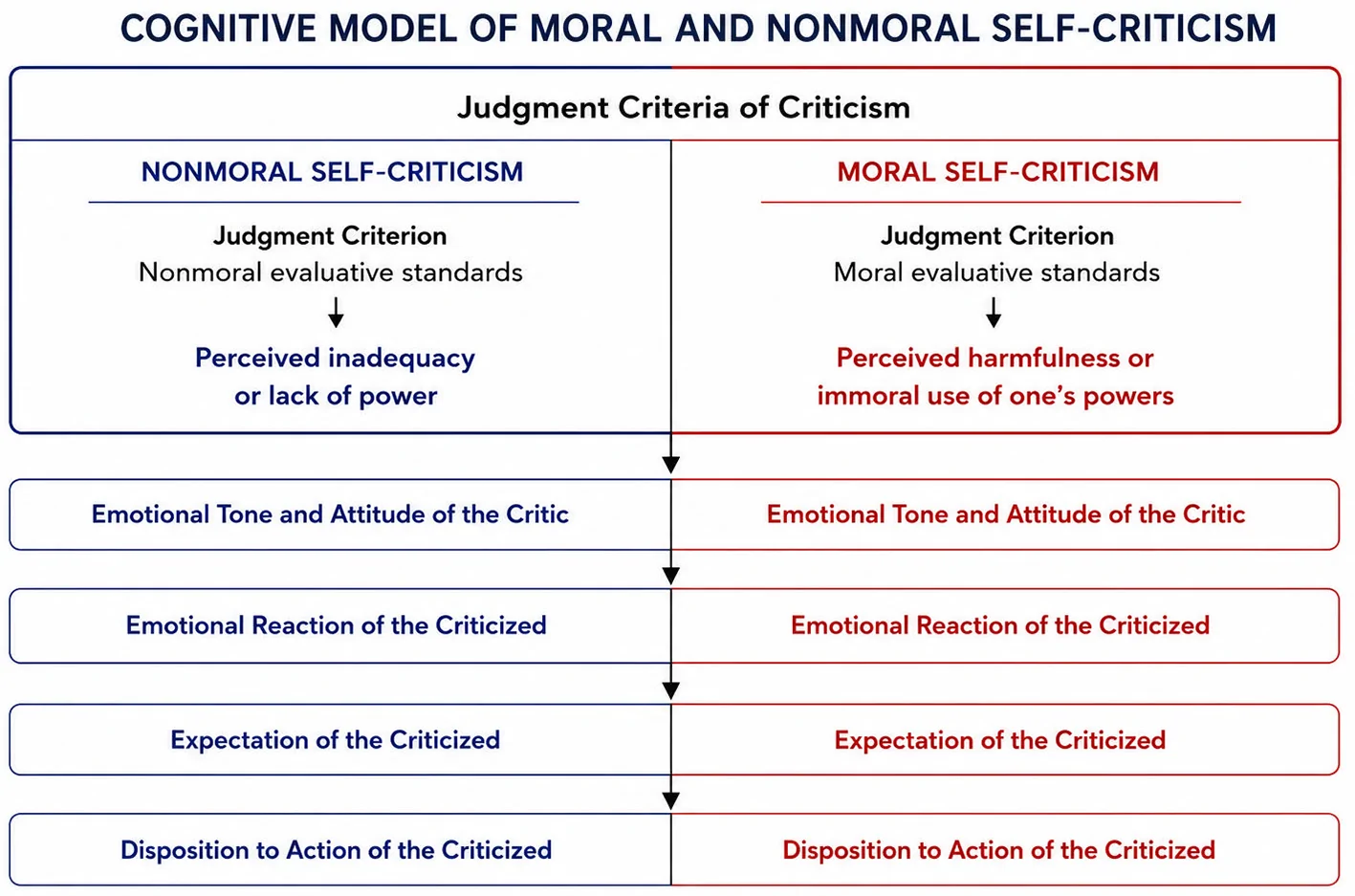
Conceptual cognitive model of moral and nonmoral self-criticism organized by judgement criteria.

The main contribution of the present model, compared with previous theoretical accounts, lies in its focus on the judgment criteria organizing negative self-evaluation, an aspect that we consider fundamental for understanding SC. Building upon this distinction, we introduce two forms of SC, namely moral and nonmoral SC, each organized by a different judgment criterion. Consistent with the components identified in previous theoretical models (Zaccari et al., 2024a), we propose that these two forms of SC are characterized by distinct configurations of the judgment criterion, emotional tone and attitude of the critic, emotional reaction, expectation of the criticized, and disposition to action (behavioral reactions) (see Figure 1). Our proposal is not intended to replace existing conceptualizations but rather to introduce the judgment criterion as an additional cognitive component contributing to the organization of self-critical emotional and behavioral experiences.

Within the proposed framework, these components are not independent. Rather, they constitute an integrated cognitive-affective sequence. Specifically, the judgment criterion through which individuals evaluate themselves is hypothesized to organize the emotional tone adopted by the inner critic, which, in turn, is associated with characteristic emotional reactions, the expectation of the criticized, and distinct behavioral dispositions. Thus, the different forms of SC described in the present framework emerge from coherent configurations of these interrelated components rather than from isolated psychological processes.

The following sections describe each component of the proposed model in detail and illustrate how they jointly characterize moral and nonmoral forms of SC, building upon the theoretical rationale developed in the preceding sections.

#### 2.1.1 Judgment criteria of self-criticism and typical inner dialogues

This theoretical perspective proposes an evaluative process that distinguishes between negative self-evaluations of *inadequacy* or *powerlessness* about oneself with respect to nonmoral standards (i.e., referring to criteria such as physical appearance, intelligence, ability, amiability, inclusion—interpersonal and socioemotional skills—and physical prowess) and self-evaluation of *harmfulness* in regard to moral standards (i.e., referring to altruistic criteria such as participation, closeness, and responsibility and deontological criteria such as *harmfulness*, duty, and wickedness) with respect to both the performance (emotions, conduct, behavior, thoughts, actions, omissions, and intentions) and competence of the self (global judgment).

In our perspective, the term “power” follows the usage proposed by Miceli and Castelfranchi (2018), where it does not refer to social or interpersonal dominance, but rather to one's abilities, resources, skills, or personal characteristics relevant to acting effectively—that is, one's sense of agency or competence. The distinction between “lack of power” and “immoral use of one's powers” therefore reflects two different evaluative domains: (a) self-deficiency or incompetence, arising from the perceived absence of the abilities, resources, or characteristics required to meet a given standard, and (b) moral failure or transgression, arising from the perceived possession of the power to violate a moral standard.

Importantly, the term nonmoral does not mean *immoral*; rather, it refers to a self-critical dialogue that is not focused on responsibility or harm (Miceli and Castelfranchi, 2018).

Building upon these two judgment criteria, we propose two forms of SC, namely nonmoral and moral. Criticism can be related to inadequacy or incompetence (criticism according to nonmoral criteria for a lack of power: lacking attractiveness, intelligence, or skills) or to issues related to immoral use of one's abilities and of one's own capacities (criticism according to moral criteria for immoral use of power: possessing the ability and willingness to violate moral rules or harm others), establishing a distinction between the evaluation of the self as stupid or deficient and the perception of the self as harmful and malicious (i.e., as evil, unjust, sinful—that is, having the power to violate moral rules and being willing to do so) (Castelfranchi, 1988; Castelfranchi and Parisi, 1980; Conte and Castelfranchi, 1995).

From this perspective, SC may therefore be directed toward two qualitatively different representations of the self: the deficient or inadequate self, organized around nonmoral judgment criteria, or the immoral or bad self, organized around moral judgment criteria.

An example of inner criticism for inadequacy or powerlessness with respect to nonmoral standards could be: “*You have no character, you have no quality, you don't know how to express yourself, you are incapable, you are boring, you can't do it, you will never be successful, you will never be satisfied, you are not capable, you are not up to it, you have no talent, you're nothing special, you won't have affection, you're ugly, you're fat, you're awkward*.”

In contrast, prototypical criticism for harmfulness or malice with respect to altruistic (i.e., referring to participation, closeness, and responsibility) moral standards could be: “*You could have helped him and you didn't, you could have been there and you weren't, you didn't do enough (for the other), you didn't show closeness to him; what did you do to deserve this luck, compared to him, you left him alone in suffering, you did nothing to help him, look now at how he suffers*.” Complementarily, prototypical criticism for harmfulness or malice with respect to deontological (i.e., referring to power and duty) moral standards could be: “*You're unforgivable, how dare you, who do you think you are... look at what a scoundrel you are, you're a wicked and disgusting person, you shouldn't have thought such things, how could you think such things, don't you realize, it's your fault that you're like this, you're a superficial, neglectful person, you must be careful otherwise you'll make unforgivable mistakes, you'll let important things slip away*.”

The present proposal should not be interpreted simply as introducing a new taxonomy of SC. Rather, it advances a broader theoretical claim concerning the cognitive structure underlying self-critical evaluation. The distinction between moral and nonmoral SC is therefore not the starting point of the model, but the consequence of the more fundamental role attributed to judgment criteria in organizing self-critical evaluation. This shift in perspective moves the discussion beyond the classification of different forms of SC toward a more fundamental analysis of the cognitive architecture underlying self-critical evaluation.

#### 2.1.2 Emotional tone and attitude of the critic

In line with previous theoretical models highlighting the importance of the emotional tone of the inner critic, we assume that internal critical voices may differ in the emotional tone and attitude they convey. Since the critical internal dialogue is viewed as one part of the self conveying critical messages to another part of the self, we consider it important to explore how individuals perceive and represent the attitude of their inner critic.

Within the present framework, moral and nonmoral forms of SC are hypothesized to differ in the emotional tone and attitude adopted by the inner critic, reflecting the different judgment criteria organizing self-evaluation. Consistent with Gilbert's model (Gilbert and Simos, 2022), particularly maladaptive forms of SC are characterized by a hostile emotional tone, reflecting activation of the threat system rather than adaptive self-correction. We propose that hostility may characterize both moral and nonmoral SC; however, its phenomenological quality and function differ according to the judgment criterion organizing self-evaluation.

Nonmoral SC, organized around nonmoral judgment criteria, may be characterized by an inner critic adopting a disqualifying, ridiculing, humiliating, demeaning, and, in more maladaptive forms, hostile tone directed toward the deficient or inadequate self. The purpose of this criticism is to emphasize perceived incompetence or inadequacy relative to the nonmoral ideal self (e.g., intelligence, ability, competence, attractiveness, physical appearance, personal worth, physical prowess, or social abilities).

Conversely, moral SC, organized around moral judgment criteria (altruistic or deontological), may be characterized by an inner critic adopting a condemning, blaming, guilt-inducing, accusatory, contemptuous, angry, and disgusted tone and, in particularly maladaptive forms, an overtly hostile attitude directed toward the immoral or bad self. This internal dialogue resembles a threatening moral warning or an uncompromising internal prosecutor, constantly warning the self about perceived responsibility, moral transgression, or the possibility of causing harm. It is worth noting that, in its most extreme forms, a hostile tone may characterize both nonmoral and moral SC, resembling what Gilbert (2022) describes as a hostile stance toward the self.

#### 2.1.3 Emotional reaction of the criticized

Within the present framework, moral and nonmoral forms of SC are hypothesized to be associated with different predominant emotional reactions, reflecting the different judgment criteria organizing self-critical evaluation. Moral SC, organized around moral judgment criteria, is more likely to be associated with emotions such as deontological or altruistic guilt, regret, or moral disgust.

Conversely, nonmoral SC, organized around nonmoral judgment criteria, is more likely to be associated with shame, embarrassment, feelings of inadequacy, or anger.

This proposal does not imply a one-to-one correspondence between forms of SC and emotional reactions. Rather, it suggests that moral and nonmoral SC, by virtue of the different judgment criteria underlying them, are more likely to organize different predominant emotional configurations. At the same time, emotions such as anxiety, sadness, frustration, or distress may accompany both forms of SC, although they are hypothesized to emerge within different evaluative organizations and to assume different psychological meanings depending on the underlying judgment criterion.

#### 2.1.4 Expectation of the criticized

We hypothesize that the emotional reaction of the criticized is associated with characteristic expectations, namely the anticipated interpersonal consequences of self-evaluation.

These expectations do not merely concern anticipated social outcomes but reflect the interpersonal meaning attributed to one's self-critical evaluation.

Nonmoral SC, organized around nonmoral judgment criteria, is more likely to be associated with expectations of passive interpersonal consequences, such as being neglected, devalued, abandoned, rejected, or socially excluded because of one's perceived inadequacy. These expectations are typically accompanied by emotional reactions such as shame and feelings of inferiority.

Conversely, moral SC, organized around moral judgment criteria, is more likely to be associated with expectations of active interpersonal consequences, including blame, moral condemnation, ostracism, or punishment. These expectations are consistent with research on guilt, moral emotions, and moral identity (Mancini and Gangemi, 2021).

The proposed model suggests that moral and nonmoral forms of SC are characterized by qualitatively different expectations of the criticized, reflecting the distinct judgment criteria through which self-critical evaluation is organized. Specifically, nonmoral SC is associated with expectations of passive exclusion resulting from perceived inadequacy, whereas moral SC is associated with expectations of active blame, condemnation, ostracism, or punishment resulting from perceived moral transgression.

#### 2.1.5 Disposition to action of the criticized

In this model, moral and nonmoral forms of SC are hypothesized to be associated with different dispositions to action (behavioral reactions), reflecting the distinct emotional reactions and expectations of the criticized organized by different judgment criteria.

Nonmoral SC, organized around nonmoral judgment criteria, is more likely to be associated with shame, embarrassment, and feelings of inadequacy, which typically motivate withdrawal, concealment, or increased efforts to build or restore one's aspired-to identity. These dispositions may give rise to coping behaviors such as avoidance, submissive strategies, perfectionistic striving, hyper-compensatory behaviors, self-punitive and self-destructive behaviors (i.e., directed toward attacking or devaluing the deficient or inadequate self), or other efforts aimed at reducing the discrepancy between the actual self and the nonmoral ideal self.

Conversely, moral SC, organized around moral judgment criteria, is more likely to be associated with deontological or altruistic guilt, regret, or moral disgust, which typically motivate a desire for reparation, expiation, or restoration of the moral ideal self (e.g., by purifying the self). These action tendencies may be expressed through behaviors aimed at alleviating the perceived moral need arising from the appraisal of having violated moral standards, including ritualistic behaviors, reassurance seeking, confession, repetitive activities, reparative actions, self-punitive behaviors (i.e., aimed at expiation or restoration of the moral ideal self), altruistic engagement, or voluntarily lowering one's own fortunes.

Accordingly, the proposed model suggests that the different behavioral tendencies described in previous theoretical models may be understood as distinct action dispositions emerging from the interaction between judgment criteria, emotional reactions, and the expectations of the criticized, rather than as independent manifestations of SC.

Notably, self-punitive behaviors may occur in both moral and nonmoral forms of SC. However, within the proposed framework, they are hypothesized to be organized by different judgment criteria and therefore to serve different psychological functions. In nonmoral SC, self-punishment primarily reflects responses to perceived inadequacy and failure to attain the nonmoral ideal self, whereas in moral SC it primarily reflects attempts at expiation, atonement, or restoration of the moral ideal self following the perceived violation of moral standards. All the components described above are summarized in Table 1.

**Table 1:** Theoretical components of the cognitive model of moral and nonmoral self-criticism.

Component	Definition	Nonmoral self-criticism	Moral self-criticism
Judgment criteria of criticism	Primary organizing cognitive component. Evaluative standards through which individuals judge themselves.	Nonmoral evaluative standards: physical appearance, intelligence, ability, amiability, inclusion (interpersonal and socioemotional skills), and physical prowess. Evaluation: perceived inadequacy or lack of power relative to the nonmoral ideal self.	Moral evaluative standards: altruistic (participation, closeness, and responsibility) and deontological (harmfulness, duty, and wickedness). Evaluation: perceived harmfulness or the immoral use of one's powers relative to the moral ideal self.
Emotional tone and attitude of the critic	Emotional quality and attitude adopted by the inner critic, reflecting the activated judgment criterion.	Disqualifying, ridiculing, humiliating, and demeaning.	Condemning, blaming, guilt-inducing, accusatory, contemptuous, angry, and disgusted.
Emotional reaction of the criticized	Predominant emotional responses associated with the activated judgment criterion.	Shame, embarrassment, feelings of inadequacy, inferiority, and anger. Anxiety, sadness, frustration, or distress may also occur.	Deontological guilt, altruistic guilt, regret, or moral disgust. Anxiety, sadness, frustration, or distress may also occur.
Expectation of the criticized	Refers to expectations about others' responses and the anticipated social and interpersonal consequences associated with self-evaluation.	Passive interpersonal consequences: neglect, devaluation, abandonment, rejection, or social exclusion.	Active interpersonal consequences: blame, moral condemnation, ostracism, or punishment.
Disposition to action of the criticized	Characteristic behavioral tendencies emerging from the interaction between judgment criteria, emotional reactions, and expectations.	Withdrawal, concealment, avoidance, submissive strategies, perfectionistic striving, hyper-compensatory behaviors, self-punitive and self-destructive behaviors aimed at reducing the discrepancy with the nonmoral ideal self.	Reparation, expiation, restoration of the moral ideal self, ritualistic behaviors, reassurance seeking, confession, repetitive activities, reparative actions, altruistic engagement, voluntarily lowering one's own fortunes, and self-punitive behaviors aimed at expiation or restoration of the moral ideal self.

## 3 Additional parameters for assessing adaptive vs. maladaptive self-criticism

### 3.1 Additional parameters of adaptive and maladaptive self-criticism

Having outlined the main components of the proposed model, it is necessary to address the following central question: when is SC, in its two forms, more likely to become maladaptive and contribute to psychopathology? From this perspective, both moral and nonmoral forms of SC may manifest in either adaptive or maladaptive ways, depending not only on their underlying judgment criteria but also on additional characteristics of the self-critical process. To complement the proposed model, we suggest considering three additional parameters that capture distinct aspects of the self-critical process: (a) its quantitative characteristics (i.e., frequency, intensity, and persistence), (b) the metacognitive factor of critical distancing, and (c) the structural organization of self-critical evaluations (i.e., their stability vs. contingency and their tendency to generalize to the individual's global self-concept). Together, these parameters help distinguish adaptive from maladaptive expressions of both moral and nonmoral SC.

### 3.2 Intensity, frequency, and persistence

The maladaptive expression of SC depends not merely on its presence, but on its frequency, intensity, and persistence.

Consistent with dimensional approaches to psychopathology, it is essential to study the “psychology of the normal” to differentiate functional from dysfunctional forms of SC. Psychological processes are often part of normal functioning but become clinically significant when they intensify or persist over time (Mancini and Gangemi, 2024).

Moderate self-critical tendencies can serve adaptive functions, fostering personal growth, responsibility, and reparative motivation when discrepancies arise with one's nonmoral or moral ideal self. However, when SC becomes chronic and persistent, it may lead to maladaptive patterns, characterized by frequent behavioral consequences and persistent painful emotional reactions that ultimately hinder daily functioning. These maladaptive expressions often include, for example, compulsive or ritualized behaviors driven by guilt, and avoidance patterns motivated by fear of moral failure or damage to self-image. In such cases, SC shifts from a regulatory process to a source of psychological distress, acting as a psychopathological maintenance mechanism. Consequently, SC has become a core therapeutic target across several psychotherapeutic frameworks (i.e., Compassionate Mind Training, Compassion-Focused Therapy, Schema Therapy, Chairwork, and Emotion-Focused Therapy; Arntz, 2018; Gilbert, 2014; Gilbert and Procter, 2006; Gilbert and Simos, 2022; Greenberg, 2011; Pugh, 2019; Young et al., 2003).

### 3.3 Critical distancing

An additional cognitive factor that may significantly modulate the impact of SC is the subjective credibility attributed to one's own critical thoughts. The individual's level of distress—and consequently, the severity of SC—is expected to increase in proportion to the degree of subjective credibility attributed to self-critical judgments (i.e., the extent to which individuals believe that what they criticize about themselves is true, useful, and deserved). This view is consistent with current theoretical perspectives emphasizing that high levels of SC tend to foster global and definitive self-judgments (Shahar, 2015), leading individuals to believe that their SC is helpful or deserved, and therefore that being harsh toward themselves is necessary and justified (Gilbert, 2022).

A high degree of subjective credibility in one's self-critical thoughts contributes to the maladaptive expression of SC, resulting in psychopathological manifestations. The more individuals perceive their self-critical evaluations as true, deserved, and useful, the more likely these evaluations are to consolidate into rigid, global, and enduring negative self-judgments, thereby amplifying psychological suffering, fueling psychopathology, and increasing resistance to therapeutic change (Gilbert, 2022; Shahar, 2015).

From our perspective, individuals may appraise their SC in different ways—seeing it as right, truthful, logical, or useful, or conversely as unfair, wrong, false, useless, harmful, or undeserved. Such an appraisal indicates a greater capacity for psychological distancing from self-critical thoughts and, consequently, a reduced negative impact on wellbeing.

### 3.4 Stability and contingency of self-critical evaluations

Among the components listed above, the degree of stability or contingency in self-critical evaluations (Fiske and Taylor, 1991) plays a crucial role in determining their severity and maladaptive potential. When SC is contingent—arising in specific contexts or in response to particular failures, perceived inadequacies, or moral transgressions—it may serve adaptive regulatory functions, facilitating self-correction and personal growth. Conversely, when self-critical evaluations become stable, they tend to generalize beyond the situations in which they originated, becoming integrated into the individual's global self-concept and leading to chronic self-devaluation, persistent painful emotional reactions, and greater functional and clinical impairment.

## 4 Discussion

### 4.1 The proposed model and its implications

The present work proposes that the judgment criterion through which the self is evaluated constitutes an additional cognitive component of the self-critical process. Within this framework, qualitatively different judgment criteria organize distinct forms of self-critical evaluation, giving rise to moral and nonmoral forms of SC. Thus, the principal contribution of the proposed model does not simply lie in distinguishing between moral and nonmoral forms of SC, but in identifying the judgment criterion as the cognitive principle through which these different forms are organized. By making explicit a cognitive component that has remained largely implicit within previous conceptualizations, the proposed model offers a more integrated framework for understanding the heterogeneity of self-critical experiences. This perspective has important implications for current psychopathological models and opens new directions for theoretical, empirical, and clinical research. The following sections discuss these implications and outline the main directions for future research.

### 4.2 Theoretical implications for psychopathology and clinical relevance

Clinically, it is evident that patients criticize and judge themselves according to different evaluative standards (e.g., moral vs. nonmoral judgment criteria), which lead to distinct emotional outcomes, coping strategies, and behavioral tendencies. Understanding these distinctions therefore appears to be essential for refining current psychopathological models. The proposed model suggests that different emotional tones of criticism, specific emotional reactions, and distinct action tendencies—when organized around moral or nonmoral judgment criteria—may contribute to different clinical phenotypes.

For instance, moral SC—based on moral judgment criteria—seems particularly relevant in disorders where guilt and responsibility play a central role, such as obsessive–compulsive disorder (OCD) and certain forms of depression.

As illustrated by the clinical vignettes presented in Section 1.8, individuals with OCD frequently experience deontological guilt arising from the fear of violating moral rules or causing harm (Gangemi and Mancini, 2017; Mancini and Gangemi, 2021; Mancini et al., 2021). Their internal dialogue often resembles a mental tribunal of self-condemnation, characterized by moral evaluations such as “I am bad,” “evil,” or “disgusting” (Dean and Purdon, 2021; Mancini, 2018; Pace et al., 2011; Saliani et al., 2024). This form of moral SC, marked by guilt and self-disgust, is typically triggered by intrusive thoughts, perceived moral failures, or the fear of having caused irreparable harm. Compulsive behaviors can thus be interpreted as reparative attempts aimed at restoring the moral ideal self.

Similar self-critical dynamics have been observed in certain forms of depression, in which altruistic guilt predominates—linked to the perception of having failed in acts of care or compassion, or of having harmed others (Basile et al., 2011; Gangemi and Mancini, 2017; Mancini et al., 2021, 2022; O'Connor et al., 2012).

In contrast, nonmoral SC, focused on inadequacy and incompetence, seems more characteristic of conditions in which self-image or perceived competence is threatened (Warnock-Parkes et al., 2022; Werner et al., 2012). For instance, in social anxiety disorder, criticism tends to be nonmoral, centered on perceptions of incompetence or inadequacy (e.g., intelligence, ability, or performance). Such evaluations elicit shame, anxiety, and embarrassment (Warnock-Parkes et al., 2022; Werner et al., 2012), which in turn motivate avoidance behaviors or perfectionistic striving aimed at restoring the nonmoral ideal self (Miceli and Castelfranchi, 2018).

Notably, although the model is likely to illuminate specific clinical conditions, it is also potentially useful for understanding transdiagnostic mechanisms and psychopathological comorbidity. Importantly, this conceptualization does not imply that the two forms of SC are mutually exclusive; rather, individuals may show a predominance of one judgment criterion over the other. Multiple evaluative standards may coexist within the same individual—and even within the same self-critical episode—although one judgment criterion is expected to play a more prominent role in organizing the emotional tone of the critic, the emotional reactions of the criticized self, associated expectations, and the resulting behavioral tendencies. The predominance of one judgment criterion over another may therefore contribute to the variability of emotional responses, behavioral tendencies, and clinical presentations observed across individuals and psychopathological conditions. At the same time, the coexistence of multiple evaluative standards may help explain both the transdiagnostic nature of SC and its overlap across different psychopathological conditions.

Beyond these specific clinical illustrations, the proposed model may also provide a useful theoretical framework for understanding more complex psychopathological conditions, such as personality disorders. However, given the marked heterogeneity of personality pathology and the multiplicity of mental states that characterize these conditions, directly associating specific judgment criteria with individual personality disorders would, at this stage, be premature and overly speculative. Rather, we propose that the distinction between moral and nonmoral judgment criteria may be more appropriately investigated at the level of self-representations and mental states (Dimaggio and Semerari, 2003), rather than at the level of diagnostic categories.

This perspective is consistent with contemporary dimensional approaches to psychopathology (American Psychiatric Association, 2022), which emphasize underlying psychological processes rather than categorical diagnoses. Likewise, several theoretical traditions have described qualitatively different self-representations that may constitute promising contexts for investigating the role of judgment criteria. For example, Kernberg (1975) described the *bad self*, characterized by representations of the self as profoundly wrong, monstrous, insane, degraded, bad, or morally unacceptable, whereas Beck et al. (1990) described self-schemas organized around beliefs such as “*I am fundamentally unacceptable*” and “*I am weak and vulnerable*.” Similarly, the mental states model proposed by Dimaggio and Semerari (2003) emphasizes that personality pathology is characterized by the coexistence of multiple problematic mental states, each associated with distinct representations of the self, others, and interpersonal relationships. Although these models derive from different theoretical traditions, they converge in describing qualitatively different ways in which individuals experience and represent themselves.

From this perspective, the concept of *judgment criterion* may represent a further theoretical lens for understanding how qualitatively different self-representations may be associated with different forms of self-critical evaluation. Exploring this possibility may contribute to explaining both the heterogeneity and the transdiagnostic nature of self-critical experiences. More generally, different judgment criteria may coexist within the same individual not only across psychopathological conditions but also in the general population, contributing to the variability of everyday self-critical experiences. However, specifying how particular judgment criteria relate to specific self-representations or clinical conditions falls beyond the scope of the present work and represents an important direction for future theoretical and empirical research.

### 4.3 Working hypotheses and future research directions

The present work constitutes a theoretical proposal designed not only to provide an explanatory framework for understanding different forms of SC, but also to generate empirically testable hypotheses and guide future research. Specifically, the proposed model predicts that self-critical experiences organized by different judgment criteria will systematically differ in their emotional, motivational, behavioral, and psychopathological correlates. Future studies should aim to empirically validate the proposed model using mixed methods, combining qualitative, quantitative, and phenomenological approaches applied to both clinical and community samples.

Three main directions can be delineated for the empirical validation and extension of the proposed model, integrating evaluative and psychopathological perspectives.

### 4.4 Phenomenological investigations of self-criticism

The use of qualitative methodologies (Zaccari et al., 2024b) may offer significant potential for deepening our understanding of self-critical mechanisms. To this end, researchers could employ narrative protocols and guided self-reflection tasks, asking participants to describe a self-critical episode, including their internal dialogue. The goal would be to examine whether moral and nonmoral forms of SC represent distinct manifestations of the phenomenon, each characterized by specific cognitive and emotional subjective experiences.

### 4.5 Development of an assessment instrument

A second line of research could focus on developing and validating a self-report instrument based on the proposed model, designed to measure the judgment criteria that guide self-critical processes (moral vs. nonmoral), as well as the frequency, intensity, and content of criticism. Additional items could assess emotional correlates (e.g., guilt vs. shame) and action tendencies (e.g., reparative vs. avoidant behaviors/conduct). Such an instrument would enable a systematic evaluation of the cognitive, emotional, and behavioral components of SC, facilitating the differentiation between adaptive and maladaptive forms and the assessment of their intensity across both clinical and nonclinical populations.

### 4.6 Associations with psychopathology

A third research direction could aim to investigate the relationships between the two forms of SC, their associations with psychological variables, and specific clinical profiles. Based on the theoretical framework proposed here, moral SC may be expected to show stronger associations with psychopathological conditions in which guilt and responsibility are prominent (e.g., obsessive–compulsive disorder), whereas nonmoral SC may be more closely linked with conditions characterized by concerns about inadequacy, competence, and self-evaluation (e.g., social anxiety disorder).

Quantitative methodologies could enable an exploration of moral and nonmoral judgment criteria and their relationships with specific psychopathological dimensions. Clarifying these associations could contribute to refining existing psychopathological models and elucidating how different evaluative criteria participate in dysfunctional self-evaluation processes.

From an assessment perspective, future research should also examine whether evaluating judgment criteria alongside broader dimensions of psychological functioning, such as metacognitive functioning (Semerari et al., 2012, 2014; Pellecchia et al., 2015), may provide a more comprehensive understanding of self-critical processes while supporting more accurate case formulation across different psychopathological conditions.

## 5 Conclusion

The present theoretical proposal has placed at its center the judgment criterion through which individuals evaluate themselves, a component that previous theoretical models had largely left unexplored.

This conceptualization would be particularly useful within the cognitive theoretical perspective, consistent with the theoretical matrix underlying the proposed model. Foregrounding this component would allow moral and nonmoral forms of SC to be explained not as isolated categories lacking a common explanatory principle, but as qualitatively distinct configurations, each organized by a different judgment criterion, the nature of which could help clarify the heterogeneity of self-critical experiences.

More specifically, the distinction between moral and nonmoral judgment criteria provides a theoretical basis for explaining why SC may assume qualitatively different emotional reactions, behavioral tendencies, emotional tones of the critic, and expectations of the criticized, while remaining expressions of the broader self-critical process.

Beyond extending current theoretical models of SC, the proposed framework would offer a novel perspective for understanding how different evaluative standards shape the phenomenology, adaptive and maladaptive manifestations, and transdiagnostic nature of SC. From a clinical perspective, identifying the judgment criterion organizing an individual's self-critical experience may represent an important target for both case formulation and psychotherapy. In line with recent developments in interventions targeting self-critical processes and promoting greater psychological distance from SC (Saliani et al., 2024), the proposed framework suggests that treatment may benefit not only from reducing the intensity of self-critical thoughts but also from progressively modifying the underlying judgment criterion. Such changes may, in turn, produce cascading effects on the emotional tone of the inner critic, the emotional reactions of the criticized self, associated expectations, and action tendencies, thereby contributing to the reduction of maladaptive coping strategies involved in the maintenance of psychological distress. The research directions outlined could contribute to refining psychopathological models, guiding the development of assessment instruments, and informing more targeted psychotherapeutic interventions tailored to the judgment criteria organizing self-critical experience. Future empirical research will be essential to validate the proposed model, clarify the role of judgment criteria in the development and maintenance of psychopathology, and further bridge the gap between theoretical conceptualization and clinical practice.

## Data Availability

The original contributions presented in the study are included in the article/supplementary material, further inquiries can be directed to the corresponding author.
